# Spatiotemporal molecular imaging is a critical part of spatiotemporal molecular medicine

**DOI:** 10.1002/ctm2.347

**Published:** 2021-03-04

**Authors:** Xiangdong Wang, Jia Fan

**Affiliations:** ^1^ Department of Pulmonary and Critical Care Medicine Zhongshan Hospital Institute for Clinical Science Shanghai Institute of Clinical Bioinformatics Shanghai Engineering Research for AI Technology for Cardiopulmonary Diseases, Jinshan Hospital Centre for Tumor Diagnosis and Therapy Fudan University Shanghai Medical College Shanghai China; ^2^ Department of Liver Surgery and Transplantation Liver Cancer Institute, Zhongshan Hospital, and Key Laboratory of Carcinogenesis and Cancer Invasion (Ministry of Education), Key Laboratory of Medical Epigenetics and Metabolism, Institutes of Biomedical Sciences, State Key Laboratory of Genetic Engineering Fudan University Shanghai China

## 1 INTRODUCTION

**FIGURE 1:**
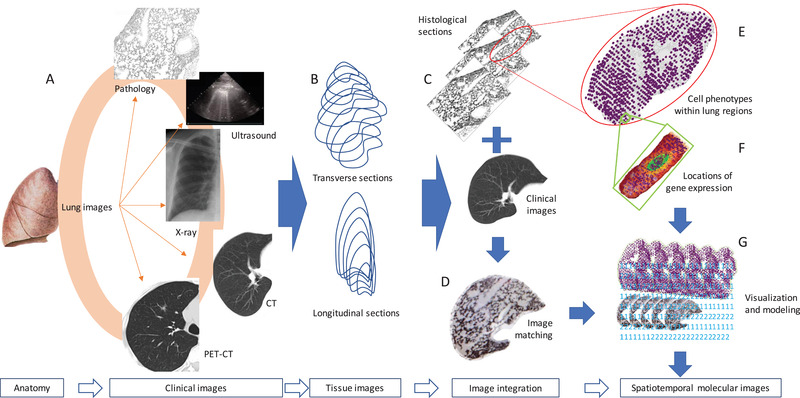
Proposed workflow of spatiotemporal molecular images. Clinical images of the organ anatomy are collected from e.g., X‐ray, computerized tomography (CT), nuclear magnetic resonance (NMR), positron emission tomography‐CT (PET‐CT), ultrasound, interventional radiology, and electrocardiogram (A). The three‐dimensional organ‐wide architectures and images can be re‐established by multiple transverse and longitudinal sections of tissues or clinical images (B). Histological sections of the organ are corresponded to clinical images (C) and then formed into an overlap by image matching (D), from which the histological structures of the organ are expected to be referred from clinical images. Spatial transcriptomes can simultaneously be performed on those histological sections in order to understand cell‐cell interactions, transcriptional signals, and phenotypes within the certain area (E) as well as mRNA expression within the cell (F). Multiple images at gene, cell, tissue, and organ levels are integrated into organ‐wide spatiotemporal molecular images using artificial intelligence, computerized programming and modeling, and visualization (G)

Spatiotemporal molecular medicine aims to emphasize the importance of integrating multi‐dimensional aspects of clinical medicine and molecular medicine ‐ “a four‐dimensional and dynamical picture of the disease by integrating clinical spatialization, temporalization, phenome, and molecular multi‐omics for disease diagnosis, therapy, and prognosis”.^1^ From an anatomical point, the “four‐dimensional” aspects were proposed to be the length, width, and height, with time of organ/tissue development in physiological and pathophysiological conditions. From a disease perspective, the four‐dimensions should include the initiation and development of disease, severity and category of disease, compliance and subjective symptoms of patients, as well as overview and manipulations from clinicians. From a diagnostic point of view, we should consider dynamic monitoring, various methodologies (e.g., real‐time polymerase chain reaction, mass spectrometer, computed tomography, pathology), biomarker quality and quantity (e.g., gene, protein, cell, image), and accuracy and repeatability. The most important issue of spatiotemporal molecular medicine is to integrate those “four‐dimensions” from various points with molecular phenotypes and to perform clinical practices at molecular levels. The present editorial addresses the importance, uniqueness, realizability, and challenges of spatiotemporal molecular images as a critical part of spatiotemporal molecular medicine. The spatiotemporal molecular image is defined as the dynamics and positioning of molecular events in clinical images (e.g., X‐ray, computerized tomography [CT], nuclear magnetic resonance [NMR], positron emission tomography‐CT [PET‐CT], ultrasound, interventional radiology, and electrocardiogram), as shown in Figure 1A. It is a comprehensive integration of clinical images, pathological morphology, and molecular profiles (e.g., genome, proteome, metabolome, and transcriptome) by principles and methodologies of clinical trans‐omics.^2^

## 2 DEFINING SPATIOTEMPORAL MOLECULAR PATHOLOGY

With spatiotemporal molecular imaging, spatiotemporal molecular pathology has been performed in experimental disease models to address gene and protein changes, cell‐cell interactions, and molecular regulations in target locations of the tissue/organ. This has not only furthered our understanding in the mechanisms of spatiotemporal cell biology and toxicology, but also given us insight into the dynamical patterns of spatial transcriptomics during the course of disease occurrence and development.^3^, ^4^ It appears hard to perform spatiotemporal molecular pathology in clinical practice, due to the dynamic availability of human tissue samples, especially samples with pathological changes, e.g., tumors, inflammation, or tissue injury. Those samples might be limited only being able to be sampled once, hard to be obtained, or ethical challenges. Maniatis et al made an outstanding exploration of spatiotemporal molecular pathology by dynamically measuring gene expression profiles of mouse spinal cord sections during the development of amyotrophic lateral sclerosis model and selectively validating critical findings of spatial transcriptome phenomes in postmortem tissue from patients with amyotrophic lateral sclerosis.^4^ This is an important approach to translate spatiotemporal molecular images into clinical practice and provides a new insight into understanding multi‐dimensional dynamics and interactions of tissue cells, e.g., among resident cells, infiltrated immune cells, or tumor cells. The routine diagnosis of clinical pathology provides critical morphological evidence and confirmation for the rapid diagnosis during operation, assistance for decision‐making in operation areas and types, the category of the disease, and target molecular heterogeneity for precision therapy. In addition, spatiotemporal molecular pathology can define cell types, locations, and communications responsible for target cell dysfunction and loss, spatiotemporal orders of gene‐gene, protein‐protein, gene‐protein interactions, pathway dynamics, inter‐cellular and intra‐pathological foci heterogeneity in a time‐, phase‐, severity‐, and nature‐dependent pattern.

## 3 PLAYING A BRIDGING ROLE BETWEEN CLINICAL IMAGES AND MOLECULAR PHENOMES

Spatiotemporal molecular pathology is a central part of spatiotemporal molecular imaging to link clinical images and molecular phenotypes. The spatiotemporal molecular imaging requests the morphological match between clinical phenomes and images, between clinical detections and microscopic structures, and between pathology and molecular omics (Figure 1). Asp et al demonstrated a strategy to map the comprehensive transcriptional landscape of cell types in the embryonic heart at three developmental stages and defined cell‐type‐specific gene expression profiles in anatomical regions of certain levels in the heart.^5^ This provides clear evidence that the three‐dimensional gene expression profiles and organ atlas can be generated along with organ anatomic architecture named “organ‐wide orchestration of gene expression.” Spatiotemporal molecular imaging enables the visualization and digital construction of transcriptome‐wide spatiotemporal patterns and multi‐dimensional expression atlas corresponding to the anatomical regions and structures. In addition to rich knowledge, experience, and methodologies, the organ‐wide orchestration of gene expression requires a massive amount of data analyses, mining, annotation, and visualization. Asp et al offered an approach to establish a database of heart spatial transcriptomics which can be further perfected in the future.^5^ The establishment of organ‐wide orchestration of gene expression will be the initial and important step to facilitate spatiotemporal molecular pathology and imaging. It requires transverse and longitudinal directions of 360^o^ sections (Figure 1B), in which more sections are measured to obtain the more precise organ‐wide atlas of gene expression (Figure 1C). The combination of multiple histological sections with clinical images provides bidirectional visualizations for the understanding of spatial image alternations, e.g., tissue histological structures corresponding to distinct constructions of computerized tomographs.

## 4 IMPROVING USES OF SPATIOTEMPORAL CLINICAL IMAGES

Spatial temporalization of clinical images has been developed and evidenced as a critical approach in clinical and translational practices, e.g., radiomics which was used to detect the alternations in the microenvironmental circulation and vascularization in solid tumors and to predict the prognosis of patients.^6^, ^7^ In addition, clinical images represent multi‐dimensional events of organs and/or tumors and can be dynamically monitored. Rizk evidenced that the dynamic three‐dimensional volume, velocity, and vascularization of the MRI‐based heart could demonstrate the quantity and quality of hemodynamics in physiological and pathophysiological conditions through adequate spatiotemporal resolution.^8^ Spatiotemporal clinical images require a large‐scale time‐dependent image dataset, with capacity of auto‐learning, mining, modeling, and assisting. With rapid development of synthetic biology and detection capacity, the panel of targeted or untargeted molecules (e.g., RNA, DNA, protein, metabolites) with strong detectable signers can directly provide real‐time multi‐dimensional clinical images. With significant improvement of technology, the reconstruction of clinical images can deliver spatiotemporal information on all organs of the body dynamically, precisely, and repeatably, including four‐dimensional ultrasound, NMR, and PET‐CT images. Qian et al recently developed the new therapeutic strategy for clinical precision medicine for multi‐organ metastasis of malignant tumor in the late phase, based on the combination of dynamic spatiotemporal four‐dimensional PET‐CT images, dynamic gene sequencing, and target‐based medications.^9^ This preliminary study presented an early proof and principle of spatiotemporal molecular medicine in clinical practice, although it was still a concept trial and does not meet the definition of spatiotemporal molecular images yet. Kevin et al demonstrated that de‐noising operator/kernel based on “HighlY constrained backPRojection” could improve the quality of spatiotemporal high frequency features, patterns, and images of reconstructed 4D composites.^10^ Those robust and accurate spatiotemporal clinical images display the potential that clinical images can be matched, covered, and corresponded with histological images (Figures 1C and 1D). This requires organ‐wide reconstruction of histological sections and clinical images with unbelievable large‐scale efforts and works.

## 5 FOCUSING SPATIOTEMPORAL FUNCTIONAL IMAGES

Spatiotemporal clinical images contribute to the routine clinical practice of diagnosis and therapy and are ready to welcome further development of organ‐wide molecular construction. Four‐dimensional ultrasound images could spatiotemporally define and characterize the qualification and visualization of protein deposition, mineral distribution, cell positioning, tissue property, and microenvironmental events, highly dependent upon ultrasound transducers, probes, and programs. Ultrasound‐oriented spatiotemporal delivery of drugs could increase the cell sensitivity to drugs and the specificity of drug‐acted locations.^11^ Clinical images enter an era of spatiotemporal multimodal organ function and construction at various layers, orientations, and aspects. For example, the integration of four‐dimensional NMR with ultrasound images, spatial cardiac electrography, and organ‐wide histological architecture or gene expression will create a new atlas with cardiac motions, electric signals, cell distributions, transcriptional networks, and signal pathways for clinicians to refer to clinical phenomes of patients to make precise decisions. Measurements and correlations of clinical images combined with other technologies can describe spatiotemporal functions of the organ, e.g., hemodynamics, motion activity, spiking activity, organogenesis, heterogeneity, and neural networks. Xu et al presented spatiotemporal dynamics of information transfer between brain regions using resting state functional magnetic resonance imaging and found the functional heterogeneity of connections between brain regions, using prediction correlation techniques.^12^ Different from spatiotemporal clinical images, spatiotemporal molecular images are expected to assist clinicians in observing the re‐organization of chromatin multi‐dimensional architectures, spatial positioning of gene expression, and cell‐cell interactions from histological structures (Figures 1E and 1F), pathological alternations, cell re‐distributions, and microenvironmental heterogeneity from clinical images (Figures 1D and 1G), and spatiotemporal changes of clinical images from the body.

## 6 NEW CHALLENGES

The process from organ anatomy to spatiotemporal molecular images is developing through various phases of spatiotemporal clinical images, tissue images, and image integration (Figure 1). The methodologies involved in various phases are currently fast‐developing and will become reachable and available for clinical investigations, even though they still need to be innovated, developed, and improved for more precise clinical applications. The sensitivity, reproducibility, and standardization of those methods are critical as they need to meet the standard operation of performance according to clinical standards. The unexpectedly huge variations among germline and somatic heterogeneities, disease properties, and complexities of molecular mechanisms require stronger and more stable deep learning and programming for visualization and model creation to integrate gene and cell information with clinical images. It is important to establish an integrative database with enough capacity to maintain exact and real‐time matches between clinical images and histological sections as well as molecular phenotypes. We should focus on the innovation and development of spatiotemporal molecular image for disease‐, organ‐, or cell‐specific events, as each image requires significant consumption of time, labor, and budget involved. Zhuang highlighted that the application of spatial transcriptomics to clinical pathology should aim to understand disease pathogenesis and to improve therapies by building enough capacity of spatial data with large‐scale initiatives and high‐throughput images.^13^ Structures and phenotypes of each gene, protein, and cell per se are changed with intra‐ and inter‐cellular variations, which can increase the difficulty to be detected, monitored, and annotated and can be worsened due to the folded complexity of 3D genome structures. Sun et al reconstructed the multiscale and three‐dimensional lung architectures by overlapping about 80,000 coronal images with the micro‐optical sectioning tomography.^14^ This is an important and valuable study to initially demonstrate the high‐precision cross‐scale visualization of 3D organ‐wide lung anatomy and morphological phenotypes (e.g., airways, arteries, veins, and alveoli). This provides potential to observe and monitor spatiotemporal alternations of organ‐wide anatomy, histological cell typing and positioning, and intracellular organelles, although dynamics of such monitoring, exact detection of organelles, tracing of target molecules, and compatibility with clinical images and molecular omics are still unclear.

In conclusion, spatiotemporal molecular imaging is a critical and indispensable part of spatiotemporal molecular medicine by integrating spatiotemporal clinical images with molecular pathology. Spatiotemporal molecular pathology includes tissue section images and positioning of gene expression and cell phenotypes by visualization and modeling to bridge clinical images and molecular profiles together (Figure 1G). Although many challenges and difficulties are present, the power of spatiotemporal molecular images can be strengthened by additional values of functional images. Spatiotemporal molecular images will provide a new insight into understanding diseases and improving therapies at multiple layers, orientations, and dimensions.
